# Hyaluronic Acid Derived from Other Streptococci Supports *Streptococcus pneumoniae In Vitro* Biofilm Formation

**DOI:** 10.1155/2013/690217

**Published:** 2013-09-19

**Authors:** Mukesh Kumar Yadav, Sung-Won Chae, Kyeongsoon Park, Jae-Jun Song

**Affiliations:** ^1^Department of Otorhinolaryngology-Head and Neck Surgery, Dongguk University Ilsan Hospital, Goyang, Gyeonggi 410-773, Republic of Korea; ^2^Department of Otorhinolaryngology-Head and Neck Surgery, Korea University College of Medicine, Seoul 135-705, Republic of Korea; ^3^Chuncheon Center, Korea Basic Science Institute, 192-1 Hyoja 2-dong, Chuncheon, Gangwon-do 200-701, Republic of Korea

## Abstract

We investigate the role of hyaluronic acid (HA) on *S. pneumoniae in vitro* biofilm formation and evaluate gene expressions of virulence and/or biofilm related genes. Biofilms were grown in medium supplied with HA derived from capsule of *Streptococcus equi*. The biomasses of biofilms were detected by crystal-violet (CV) microtiter plate assay, and the morphology was viewed under scanning electron microscope (SEM). The gene expressions were assessed by relative quantitative RT-PCR. The results showed that the HA support pneumococcal growth in planktonic form and within biofilms. The CV-microtiter plate assay detected significantly increased biofilm growth in medium containing HA. The SEM analysis revealed thick and organized biofilms in positive control and HA supplemented medium. The *nanA*, *nanB*, *bgaA*, *strH*, *luxS*, *hysA*, *ugl*, and PST-EIIA encoding genes were significantly upregulated in the planktonic cells grown in presence of HA, while the *lytA* and *comA* genes were downregulated. Similarly the *luxS*, *hysA*, *ugl*, and PST-EIIA encoding genes were significantly upregulated by more than 2-folds in HA biofilms. The results of this study indicate that the HA derived from capsule of *S. equi* supports pneumococcal growth in planktonic state and within biofilms and upregulated virulence and biofilm related genes.

## 1. Introduction

*Streptococcus pneumoniae *causes otitis media, pneumonia, meningitis, and sepsis, particularly in young children and the elderly [1, 2]. *S. pneumoniae *colonizes the human nasopharynx during the first months of life, where it can persist as part of the commensal flora. Pneumococcal colonization and disease are often associated with biofilm formation [3, 4]. Various reports have documented the presence of biofilm in the lungs of animals infected with *S. pneumoniae *[5] and in human biopsy specimens of the sinus and middle ear mucosa and the adenoids [6–8].

*S. pneumoniae* depends on carbohydrates for its growth; however, there is a low concentration of free carbohydrate in the nasopharynx, a niche where initial colonization takes place [9]. Instead, carbohydrates are found in the form of glycans and glycosaminoglycan modifications on human lipids and proteins. The most common terminal carbohydrate present on N- and O-linked glycans is sialic acid [10], which is cleaved by pneumococcal neuraminidase NanA. Previous studies have reported the upregulation of virulence related genes and increased biofilm growth in presence of glycoconjugates [11, 12].

Hyaluronic acid (HA), a glycosaminoglycan, is a key component of the extracellular matrix that is present on the apical surface of the epithelial cells [13]. Several bacterial species, including *S. pyogenes* and *S. equi*, which colonize the upper respiratory tract, express a hyaluronic acid capsule [14]. The HA derived from these bacterial capsules has essentially the same structure as that isolated from humans. Previous studies have reported that *S. pneumoniae* can utilize human and bacterial HA as a carbon source and demonstrated that hyaluronate lyase, a putative PTS transporter (PTS-EIIA), and *ugl *genes are required for pneumococcal growth on human HA [15–17]. However little is known about the role of bacterial derived HA on pneumococcal biofilms formation and expression of virulence and pathogenesis related gene. 

Therefore, we investigate the role of HA on *S. pneumoniae in vitro* biofilm formation and evaluate gene expressions of virulence and/or biofilm related genes.

## 2. Materials and Methods

### 2.1. Bacterial Strain and Growth Conditions

*S. pneumoniae* D-39 (NCTC 7466) an encapsulated, serotype 2 pathogenic strain was obtained from the Health Protection Agency Culture Collections (HPA, Salisbury, UK). Bacteria were routinely grown in tryptic soy broth (TSB) or on blood agar plates supplemented with 5% v/v sheep blood at 37°C in an atmosphere of 5% CO_2_. Yeast extract medium (YE) was prepared with 10 g/L yeast extract (Becton Dickinson), 5 g/L NaCl, and 3% wt/vol of 0.5 moles K_2_HPO_4_ [12]. *S. equi* derived hyaluronic acid was purchased from Sigma (Sigma 53747, MO, USA).

### 2.2. Effect of HA on Planktonic Cell Growth

To determine the effect of HA on planktonic cell growth, *S. pneumoniae* D39 strain was grown in YE medium or YE medium supplied with either HY or glucose (positive control). The pneumococcal colony was grown in TSB medium up to log phase, pelleted by centrifugation, and washed with phosphate buffer saline (PBS), and diluted (1 : 100) cell suspension was prepared in YE medium. Hyaluronic acid and glucose 0.2% (wt/vol) were added, and cells were incubated at 37°C in 5% CO_2_. The cell growth (0–48 hrs) was detected by measuring the optical density (OD) at 600 nm. 

For the gene expression study, the planktonic cells were grown up to log phase in corresponding medium, that is, in YE medium alone or in presence of either 0.2% HY or Glu, and immediately processed for RNA extraction. The RNA extracted from cells grown in YE medium without supplements was used as standard condition for relative quantification of gene expression.

### 2.3. Effect of HA on *In Vitro* Biofilm Growth

The effect of HA on pneumococcal biofilm growth was studied in YE medium and YE medium supplemented with 0.2% HA. Biofilms grown in YE medium supplied with 0.2% glucose was used as positive control. *In vitro* biofilm formation was carried out in 96-well (flat-bottom) polystyrene tissue culture plate (BD falcon, Sparks, MD, USA) in static model by a procedure previously described [18, 19]. The cell suspension was prepared as given above and inoculated 200 *μ*L in 96-well microtiter plate. The plates were incubated at 37°C in 5% CO_2_ for various time points (6, 12, 18, 24, and 30 hrs). After incubation, medium was discarded and plates were gently washed three times with 200 *μ*L sterile PBS. Thereafter, plates were air-dried and stained with 50 *μ*L crystal violet (CV) (0.1%) for 15 min. Excess stain was decanted off, and plates were washed three times with sterile distilled water. The biofilm was dissolved in 200 *μ*L of 95% ethanol, and OD at 570 nm was measured in an automatic spectrophotometer. All experiments were performed in triplicate, and the average was calculated. The experiments were repeated three times. 

For relative quantification of gene expression of pneumococcal biofilms, biofilms were grown for 24 and 30 hrs in a 24-well tissue culture plate by the procedure described early. The adherent cells of two wells were scraped and immediately processed for RNA extraction. The RNA extracted from biofilm cells grown in YE medium (without supplements) was used as a standard for relative quantification of gene expression. 

### 2.4. Scanning Electron Microscopic Analysis of Pneumococcal Biofilm

For SEM analysis, biofilms were grown in a 24-well tissue culture plate by the procedure described early and incubated at 37°C for 24 hrs in 5% CO_2_. After incubation, the medium was removed and the plate was gently washed two times with sterile PBS to remove planktonic cells. The SEM analysis was performed using a variable pressure field emission scanning electron microscope (VP-FE-SEM, SUPRA-55VP, Carl Zeiss, Germany). The samples were fixed with 4% formalin solution, dehydrated in a gradual ethanol/distilled water mixture from 50 to 100% in steps, and dried for 2 days. The morphologies of samples were coated with platinum (Pt) and observed with VP-FE-SEM.

### 2.5. RNA Extraction and cDNA Synthesis

For RNA extraction, biofilm cells were scraped with a sterile scraper, washed twice with sterile PBS, and immediately processed for RNA extraction. For planktonic cell RNA, 5 mL log-phase grown cells were washed twice and used for RNA extraction. Total RNA was extracted by using “RNeasy Total RNA Isolation System Kit” (Qiagen, MD, USA) according to the manufacturer's instructions with few modifications: on column RNAase-free DNAse (Qiagen, MD, USA) treatment was performed for 10 min at 20–25°C. The concentration and quality of RNA were assessed with a spectrophotometer. cDNA synthesis was carried out by using the “ImProm-II Reverse Transcriptase Kit” (Promega, WI, USA) according to the manufacturer's instructions. Tailing of RNA with a random hexamer primer was performed at 70°C for 5 min, annealed at 25°C for 5 min, extended at 37°C for 1 hr, and inactivated of samples at 70°C for 15 min.

### 2.6. Relative Quantification of Gene Expression by Real-Time PCR

Quantitative real time PCR was performed as previously described (20) with a light cycler apparatus by using the SYBR Green I Master Mix (Roche Applied Science, IN, USA). Primers were designed by standard procedures from nucleotide sequence of *S. pneumoniae* R6 strain and checked for corresponding sequence in D39 strain (Table 1). A set of 11 genes to be monitored (involve in pneumococcal biofilm formation, HA metabolism, and autolysis) were decided after reviewing the literature. Primers used for *gyrB *(spr1099) and *comA *genes (spr0043) were as previously described [20]. Real-time PCR was carried out in a total volume of 20 *μ*L, consisting of 10 *μ*L 2x SYBR Green PCR Master Mix (Roche Applied Science, IN, USA), 2 pmol of forward and reverse primers, and 2 *μ*L cDNA. PCR conditions included initial denaturation at 95°C for 10 min, followed by 45 cycles of denaturation (95°C for 10 s), annealing (56°C for 15 s), extension (72°C for 15 s), and final extension (72°C for 5 min) followed by melting curve analysis from 60 to 95°C. Negative controls containing sterile water instead of RNA were run concomitantly to confirm that the samples were free from contamination. To verify the absence of contaminating genomic DNA, each RT-PCR experiment included a no reverse transcriptase control. The relative gene expression was analyzed by using the 2^−ΔΔCT^ method [21], and the reference gene was *gyrB*. The planktonic cells grown in YE medium were used as the standard condition for the planktonic cell gene expression study. The biofilms grown in YE medium were used as the standard condition for the gene expression of cell grown within biofilms. The experiments were repeated two times in quadruplicate.

### 2.7. Statistical Analysis

Statistical analysis was performed using 2-tailed Student's test and one-way analysis of variance (ANOVA), and a difference of *P* < 0.05 was considered significant. 

## 3. Results

### 3.1. HA Support Pneumococcal Growth

**Figure 1:**
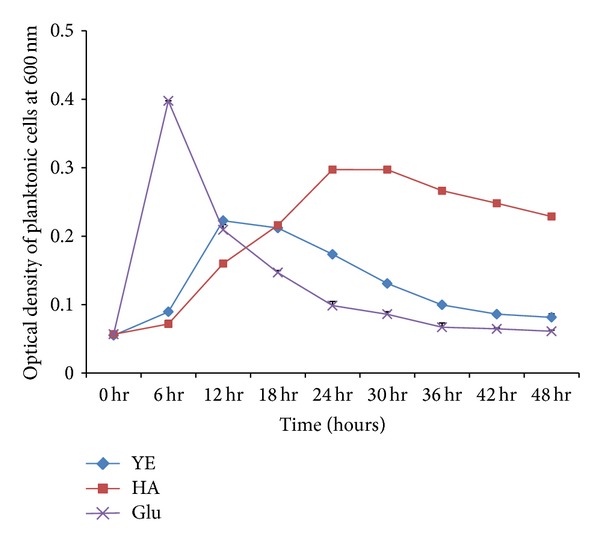
Planktonic cell growth curve of *S. pneumoniae* D-39 strain in YE medium and YE medium supplied either with 0.2% hyaluronic acid (HA) or glucose (Glu). The OD_600_ was measured at 6-hour time interval. The error bars represent standard deviations of the mean value.

Experiments performed in YE medium or in YE medium supplied either with HA or Glu showed that HA supports the growth of *S. pneumoniae* D-39 strain (Figure 1). In positive control (glucose) the bacterial growth was high, and bacteria attained log phase after 2 hours of inoculation. However, in presence of HA, bacteria attained log phase around 15 hrs of inoculation, with the doubling time greater than that of the positive control. The stationary phase was longer in HA compared to control. The OD_600_ of positive control decreased sharply at decline phase, indicating higher rate of cell lysis. However the OD_600_ of HA containing medium decreased slowly in comparison to positive control. These results indicate that probably the cell lysis process was slow in presence of HA.

### 3.2. HA Support Pneumococcal Biofilm Growth

**Figure 2:**
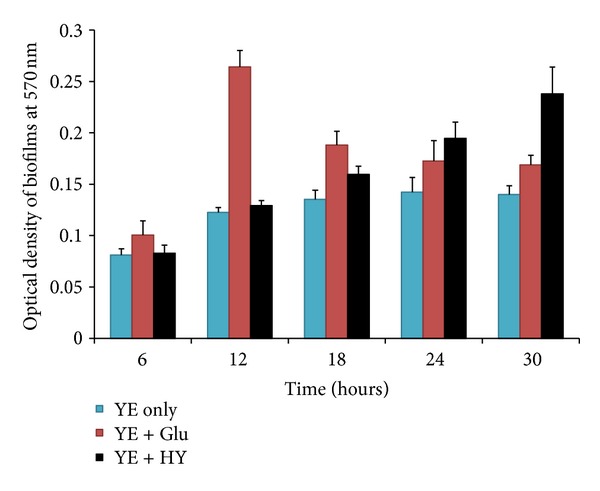
Effect of hyaluronic acid (HA) on pneumococcal biofilm growth. Time course experiment in YE medium (blue bars) or YE medium supplied with either 0.2% glucose (red bars) or hyaluronic acid (black bar). The biomass of biofilm were detected by crystal-violet microtiter plate assay. The significance of the results (*P* < 0.05) between biomass of biofilm grown in positive control (YE + Glu), hyaluronic acid (YE + HY), and negative control (YE only) was determined by one-way analysis of variance (ANOVA). The error bars represent the standard deviations (SD).

The CV microplate assay detected increased biofilm growth in medium containing HA and Glu (positive control) in compare to YE medium (Figure 2). At 6 and 12 hours of incubation, there was significant (*P* < 0.05) increase in biofilms growth in Glu medium compared to YE and HY media. However, at 18, 24, and 30 hrs of incubation, significantly increased biofilms were detected in HA and Glu media (*P* < 0.05). The biofilm biomasses of the positive control (Glu) decreased at 18, 24, and 30 hrs, in time-dependent manner. In contrast to the positive control, the biomasses of HY biofilm significantly increased at 18, 24, and 30 hours. This may be due to higher cell growth and thereafter higher cell lysis in Glu biofilms compared HY biofilms. The result indicates that HA supports *in vitro* biofilm growth of *S. pneumoniae* D39.

### 3.3. Scanning Electron Microscopic Analysis of Pneumococcal Biofilm

**Figure 3:**
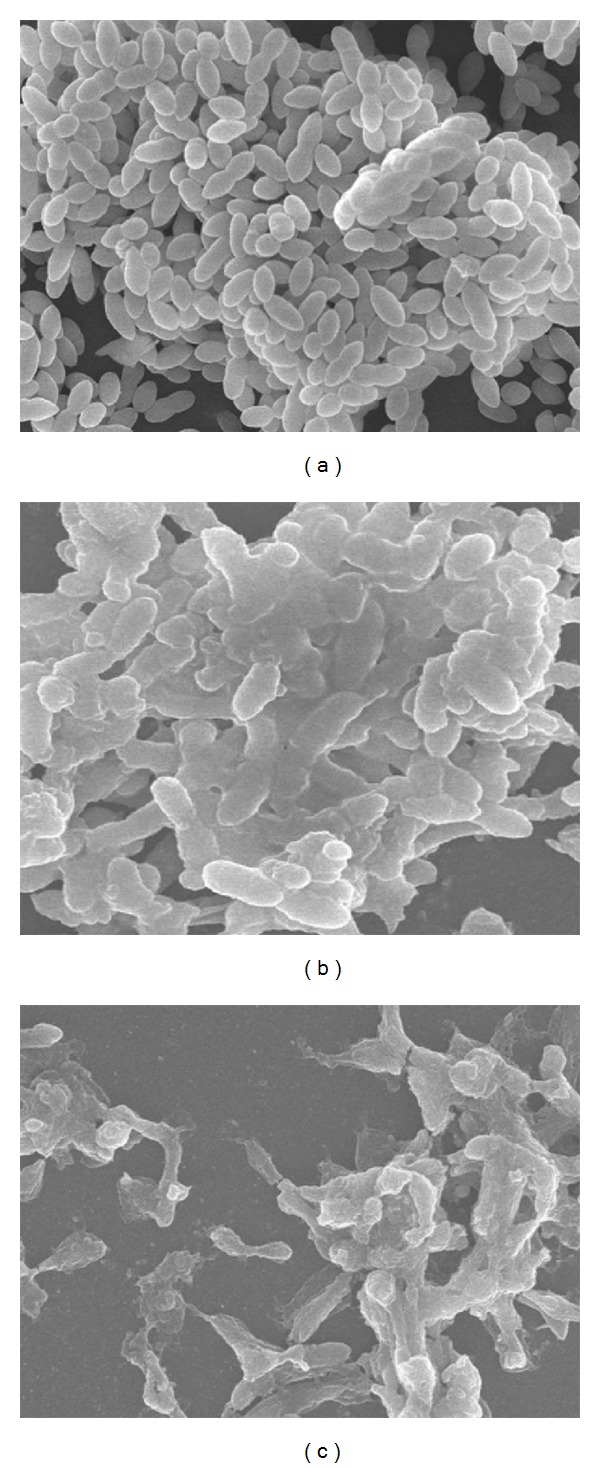
Scanning electron microscope image of *S. pneumoniae* biofilms formed in 24-well tissue culture plate. (a) Biofilm formed in YE medium supplied with 0.2 % hyaluronic acid. (b) Biofilm formed in YE medium supplied with 0.2% glucose. (c) Biofilms formed in YE medium only.

Scanning electron microscopic images of *S. pneumoniae* biofilms grown in YE medium or in YE medium either supplied with HA or Glu are shown in Figure 3. Pneumococcal biofilm growth in HA medium is thick with organised structures. The adherent cells are interconnected to each other and/or bound the cells to the intercellular matrix (Figure 3(a)). No dead cells were visible in the HA biofilms. The biofilms in positive control samples (Glu) were thick and organised, with fully grown cells, and few dead cells are visible (Figure 3(b)). No organized biofilms growth was detected in YE medium without supplements. In YE medium very few dead cells were detected, which were scattered and attached to the bottom of the tissue culture plate (Figure 3(c)).

### 3.4. Relative Quantification of Gene Expression

The fold changes in gene expression of planktonic cells grown in presence of HA or Glu in comparison to cell grown in YE medium are shown in Table 2.

The *lytA* (0.5-folds) and *comA* (0.5-folds) genes were significantly (*P* < 0.05) downregulated in planktonic cell grown with HA and upregulated in presence of glucose by 2.0- and 1.5-folds, respectively. However, the *luxS* gene was significantly (*P* < 0.05) upregulated in HA (2.1-folds) and glucose (1.5-folds) medium.

The *nanA* (3.2-folds), *nanB* (3.0-folds), *bgaA* (2.9-folds), and *strH* (2.1-folds) were significantly (*P* < 0.05) upregulated in HA medium. And in presence of glucose, the *nanA*, *nanB*, *bgaA*, and *strH* genes were upregulated by less than 2-folds (nonsignificant).

The three genes involve in HA degradations, *hysA* (2.1-folds), *ugl* (2.9-folds), and PTS-EIIA (4.0-folds) were significantly (*P* < 0.05) upregulated in presence of HA. In presence of glucose, the PTS-EIIA gene was upregulated by 3.0-folds, and the *hysA* and* ugl* genes were upregulated by less than 2-folds.

The results of relative quantification of gene expression of pneumococcal biofilm grown in HA medium with respect to biofilms grown in YE medium are shown in Table 3. The *luxS* (2.0-folds), *nanA* (2.9-folds), *hysA* (2.0-folds), *ugl* (2.8-folds), and PTS-EIIA encoding genes (2.0-folds) were significantly upregulated in HA biofilms grown for 24 hours. Similarly, in biofilms grown for 30 hours in presence of HA, the five biofilm related genes, *luxS* (2.4-folds), *nanA* (2.5-folds), *hysA *(2.3-folds), *ugl* (2.0-folds), and PTS-EIIA (2.2-folds) genes, were significantly upregulated.

## 4. Discussion 

*S. pneumoniae *can modify *N*-linked glycans, *O*-linked glycans, and glycosaminoglycans present in the human airway, and on cell surface of other streptococci. Utilization of these glycoconjugates provides a carbon source for growth, biofilm formation, competition with other bacteria within the airway, and exposing receptors for adherence. Previous studies have reported that pneumococci can utilize HA derived from human and bacteria for its growth. We studied the effect of HA on pneumococci growth in planktonic form and within *in vitro* biofilms and evaluate the changes in gene expression of virulence and/or biofilm related genes.

Our results demonstrated that HA supports pneumococcal growth in planktonic and within biofilms and upregulates the gene expression of virulence and biofilm related genes. In previous study, Marion et al. [16] reported that the *S. pneumoniae* 1121 strain can utilize *S. progenes* HA capsule for its growth and colonization. Here, our results demonstrated that *S. pneumoniae* D-39 strain can effectively utilize *S. equi* HA capsule for its growth and *in vitro* biofilms formation. The SEM analysis demonstrated thick and organised *in vitro* biofilms in presence of HA and Glu (positive control). Using *in vitro* biofilm model, we detected upregulations of virulence and biofilm related genes. The pneumococcal growth on HA derived from *S. equi* and expressions of virulence related genes indicates that *S. pneumoniae* can utilize HA capsules of other streptococci and use the carbohydrates derived from them for efficient growth and expression of virulence [22]. The modifications of capsule may make the other streptococci vulnerable to phagocytic killing by host cells, thereby decreasing interspecies competitions [23–25]. Thus it can be assumed that in *in vivo*, at niche, a site where multiple species are present, the utilization of HA capsule by pneumococci contributes to colonization both through niche competition and acquisition of carbohydrates. 

The growth of pneumococci in the presence of HA was low with longer lag phase, and the doubling time of bacteria was more than the positive control (glucose). The reduced growth efficiency of glycoconjugates was observed for N-linked glycans [12, 16, 26]. At decline phase, the rate of decrease of OD_600_ of HA culture was slow in comparison to cell grown in glucose medium (control). This might be due to slow cell lysis in presence of HA. To look further inside, we analysed the gene expression of autolysis related gene (*lytA*). Autolysis is mainly dependent on the activity of the muramidase (LytA) and on cell-wall composition [27, 28]. We detected downexpression of *lytA* gene and competence related genes (*comA*) in cells grown in HA medium. The autolysis and competence in *S. pneumoniae* are linked to *luxS* gene, which is upregulated in this study in cells grown with HA. Romao et al. [29] reported that functional *luxS* was found to be related on the one hand to downregulation of competence and on the other hand to attenuation of autolysis in cultures entering stationary phase. 

Interestingly, we detected upregulation of *nanA*, *nanB*, *bgaA*, and *strH *genes in presence of HA. The pneumococcal exoglycosidases encoding genes (*nanA*, *nanB*, *bgaA*, and *strH*) are virulence related genes that have been upregulated in presence of glycoconjugates [12, 26]. The upregulation of exoglycosidases encoding genes in presence of HA indicates that probably HA stimulates the expressions of these genes due to change in carbohydrate source. In *S. pneumoniae*, regulation of carbohydrate-specific catabolic pathways as well as modulation of virulence factors by catabolite control protein A (CcpA) has been reported [30, 31], and the alteration in carbohydrate source and metabolic pathway leads to an increase in expression of these virulence factors [32, 33]. No significant changes in neuraminidases encoding gene in glucose medium are in agreement with previous study that reported that glucose represses neuraminidase expression [34].

The upregulation of *hysA*, *ugl*, and PTS-EIIA in presence of *S. equi* capsule HA indicates that similar set of genes are required for the utilization of bacterial and human derived HA [16]. Marion et al. [16] found that the hyaluronate lyase (hyl or hysA in R6), a putative PTS transporter (PTS-EIIA), and *ugl *are required for pneumococcal growth on human HA. The surface-associated hyaluronate lyase (hyl) produced by* S. pneumoniae *is a virulence factor that degrades HA into disaccharide units [15]. These disaccharides are transported by putative PTS transporter and further cleaved by glucuronyl hydrolases (ugl) [17].

HA derived from capsule of *S. equi* supports *in vitro* pneumococcal biofilm formation. Our relative quantification results showed upregulation of *luxS*, *nanA*, *hysA*, *ugl*, and PTS-EIIA in HA biofilms. The role of *luxS* and *nanA* in pneumococcal biofilms has been reported earlier [11, 19]. A previous report demonstrated the upexpression of PTS-EIIA and *ugl *during infection of the lung, indicating that utilization of HA may also contribute to bacterial growth during disease [35]. Probably in *in vivo* the pneumococcal hyaluronate lyase aids bacterial invasion and this localized invasion contributes to bacterial colonization in addition to degrading HA for nutrition [14, 36].

In conclusion, this study indicates that the HA derived from capsule of *S. equi* supports pneumococcal growth in planktonic and within biofilms and upregulated virulence and biofilm related genes. This study will enhance our knowledge of pneumococcal pathogenicity.

## Figures and Tables

**Table 1:** Oligonucleotides designed from *Streptococcus pneumoniae* strain R6 genome and used in this study.

Gene	Locus	Primer sequences	Amplicon size in base pair
*hysA *	spr0286	TGTCGTTCGAACAGTCAGGG	120 bp
		TTGTTGATGGCACGAGGTGT	
*ugl *	spr0292	ATCACCATGATCTCGGCTTC	97 bp
		GCAGCTTTCAAGGTTGCTTC	
PTS-EIIA	spr0291	TCTCGGGTTTGAACTTAGCCA	96 bp
		GGGCCGCCACTACTGATTTA	
*nanA *	spr1536	CCAATGCTTCAAATGGTCAG	120 bp
		TATAGAATGCTGGGGCCTTG	
*nanB *	spr1531	ATTGGCTCACTCCACGTTTT	94 bp
		ATGCACGTTATGGTGGGACT	
*bgaA *	spr0565	CAAGCCAGCCGTGAACGCTATAAGG	128 bp
		GAGTGGGCAGTCAGGGTGAATTTCC	
*strH *	spr0057	GGTTTTTCCGTTGGCAGTAA	121 bp
		GCTCAAACGCATCGTAGACA	
*lytA *	spr1754	CGTCCCAGGCACCATTATCA	95 bp
		CTGGCGGAAAGACCCAGAAT	
*comA *	spr0043	GAGACGCGAGCCATTAAGG	156 bp [20]
		GGGATCTGGATCGGCAATATGA	
*luxS *	spr0308	TATGTTCGCTTGATTGGG	105 bp
		GCCGGCAGTAGGGATAGAGT	
*gyrB *	spr1099	CAGATCAAGAAATCAAACTCCAA	171 bp [20]
		CAGCATCATCTACAGAAACTC	

**Table 2:** Relative quantification of gene expression of *Streptococcus pneumoniae* planktonic cells by quantitative real-time RT-PCR.

Genes	Encoding proteins	Fold change in HA medium	*P* value	Fold changes in Glu medium	*P* value
*lytA *	N-acetylmuramoyl-L-alanine amidase	0.5	0.04	2.0	0.02
*comA *	Competence factor transporting ATP-binding/permease protein ComA	0.5	0.03	1.5	0.02
*luxS *	S-ribosylhomocysteinase	2.1	0.04	1.5	0.04
*nanA *	Neuraminidase A	3.2	0.04	1.7	0.01
*nanB *	Neuraminidase B	3.0	0.02	1.2	0.01
*bgaA *	*β*-Galactosidase precursor	2.9	0.04	1.5	0.04
*strH *	N-acetylglucosaminidase	2.1	0.02	1.6	0.04
*hysA *	Hyaluronidase/hyase	2.1	0.02	1.1	0.02
*ugl *	Unsaturated glucuronyl hydrolase	2.9	0.02	1.7	0.04
PTS-EIIA	PTS system, N-acetylgalactosamine-specific IIA component	4.0	0.04	3.0	0.03

**Table 3:** Relative quantification of gene expression of pneumococcal biofilms by quantitative real-time RT-PCR.

Genes	Fold changes in HY biofilms with respect to YE biofilms (24 hrs)		Fold changes in HA biofilms with respect to YE biofilms (30 hrs)	
	Fold changes	*P* value	Fold changes	*P* value
*luxS *	2.0	0.03	2.4	0.02
*nanA *	2.9	0.05	2.5	0.02
*hysA *	2.0	0.05	2.3	0.03
*ugl *	2.8	0.03	2.0	0.03
PTS-EIIA	2.0	0.05	2.2	0.05
